# Conspiracy beliefs and perceptual inference in times of political uncertainty

**DOI:** 10.1038/s41598-024-59434-4

**Published:** 2024-04-18

**Authors:** Salomé Leclercq, Sébastien Szaffarczyk, Pantelis Leptourgos, Pierre Yger, Alexandra Fakhri, Marielle Wathelet, Vincent Bouttier, Sophie Denève, Renaud Jardri

**Affiliations:** 1 INSERM U1172, CHU Lille, Lille Neuroscience and Cognition Centre, CURE Platform, Fontan Hospital, Lille University, 59000 Lille, France; 2 Otto-Von-Guericke Universität Magdeburg, Magdeburg, Germany; 3 LNC, INSERM U-960, Institut de Sciences Cognitives, École Normale Supérieure, 75005 Paris, France

**Keywords:** Neuroscience, Psychology

## Abstract

Sociopolitical crises causing uncertainty have accumulated in recent years, providing fertile ground for the emergence of conspiracy ideations. Computational models constitute valuable tools for understanding the mechanisms at play in the formation and rigidification of these unshakeable beliefs. Here, the *Circular Inference* model was used to capture associations between changes in perceptual inference and the dynamics of conspiracy ideations in times of uncertainty. A bistable perception task and conspiracy belief assessment focused on major sociopolitical events were administered to large populations from three polarized countries. We show that when uncertainty peaks, an overweighting of sensory information is associated with conspiracy ideations. Progressively, this exploration strategy gives way to an exploitation strategy in which increased adherence to conspiracy theories is associated with the amplification of prior information. Overall, the *Circular Inference* model sheds new light on the possible mechanisms underlying the progressive strengthening of conspiracy theories when individuals face highly uncertain situations.

## Introduction

Conspiracy theories (CTs) have drawn increased attention in the scientific community over the past few decades, and their consequential, universal, emotional and social components are at the center stage of this emerging research domain^1^. CTs are commonly defined as beliefs that assume the existence of a secret group or organization that operates maliciously and for its own benefit^2^. Adherence to multiple unrelated CTs that contradict each other is disputed^3^ yet well replicated^4–7^, which suggests the existence of common underlying mechanisms by which belief in CTs arises.

Interestingly, a first line of research has revealed that highly polarizing societal or political events might induce significant increases in stress and anxiety^8^ that can even lead to posttraumatic stress disorder symptoms^9^ or physiological changes^10–12^. Conspiratorial beliefs often crystallize around such events^13^ and may serve as coping mechanisms for dealing with stress and loss of control when uncertainty increases sharply^14–18^. Although CTs can induce widespread misconceptions—as has been observed during the COVID-19 pandemic—they also constitute intuitive explanations for complex issues (e.g., simple cause–effect relationships) that can meet people’s need to restore predictability^2^ at the cost of suboptimal reasoning.

A second line of research has focused on the role of reasoning biases in CT emergence^19–21^. According to this framework, conspiracists may bias the weight they attribute to certain stimuli to reduce uncertainty^22–24^, which sometimes leads people to aberrant salience attribution or jumping to conclusions (JTC) when they have to make probabilistic decisions. Conspiracy ideations have also been associated with a thinking style that is more intuitive^6,21^ than the common analytical approach. People who endorse CRs might tend to engage in this fast, preconscious and spontaneous processing because of specific reality-testing deficits^25^.

These results have not always been replicated, which has led some authors to wonder whether CTs can mainly be traced back to social constructs^26–28^. However, others suggest that this social learning depends on broader associative mechanisms that are responsible for the detection of predictive relationships in every natural domain^29^. This conceptualization appears to be compatible with the Bayesian framework, which assumes that cognitive and perceptual factors are rooted in a common inferential mechanism that consists of combining noisy or ambiguous sensory data with prior beliefs using the Bayes rule^30^. Thus, methods that facilitate the assessment of such probabilistic processing could provide a complementary approach to addressing the existing link between CTs and uncertainty.

Surprisingly, few attempts have been made to investigate the potential links between perceptual inference and conspiracy ideations in a controlled experimental setting. Nevertheless, some results from the CT literature appear to be compatible with a probabilistic formalism. Dagnall and colleagues^31^ explored the link between CTs and a wide range of cognitive-perceptual factors. They showed that these factors, including hallucination proneness, often conceptualized as false inferences^32^, were associated with CTs. Additionally, conspiracy ideations were found to be associated with illusory visual pattern detection^27,33^, which is a phenomenon that has regularly been explored through the prism of Bayesian theory^34^.

Very few papers have directly fitted computational models to behavioral data in nonclinical samples, with some noticeable exceptions exploring paranoia and/or conspiracy ideations^35,36^. Purely theoretical papers have also confirmed that computational approaches could help to better understand the spread of extreme beliefs including CTs on simulated or social media data^37–39^. Crucially, a more personalized computational lens^40^ and a study of CTs in their ecological environment^41,42^ seem to be needed to decipher the respective contributions of sociopolitical factors and information weighting to CT emergence.

Thus, combining the strengths of normative and ecological research during uncertain societal crises appears to be necessary for establishing a bridge between CT and inference quantification. In the present paper, we utilized *Circular inference* (CI), a Bayesian framework that has been proven effective in capturing not only JTC in patients with psychosis^43,44^ but also both perceptual^45^ and cognitive^46^ inferential suboptimality in nonclinical populations. These last results suggest that the CI framework could be suitable for capturing other variations from suboptimal inference in the general population. Based on this idea, we hypothesized that by fitting the CI model to a simple bistable task, we could benefit from using an ideal setup to challenge the potential links between (i) the inferential mechanisms at play under conditions of extreme uncertainty and (ii) the dynamics of conspiracy ideations in large populations exposed to natural sociopolitical stress.

## Results

### Measuring multilevel inference before and after stressful political events

**Figure 1:**
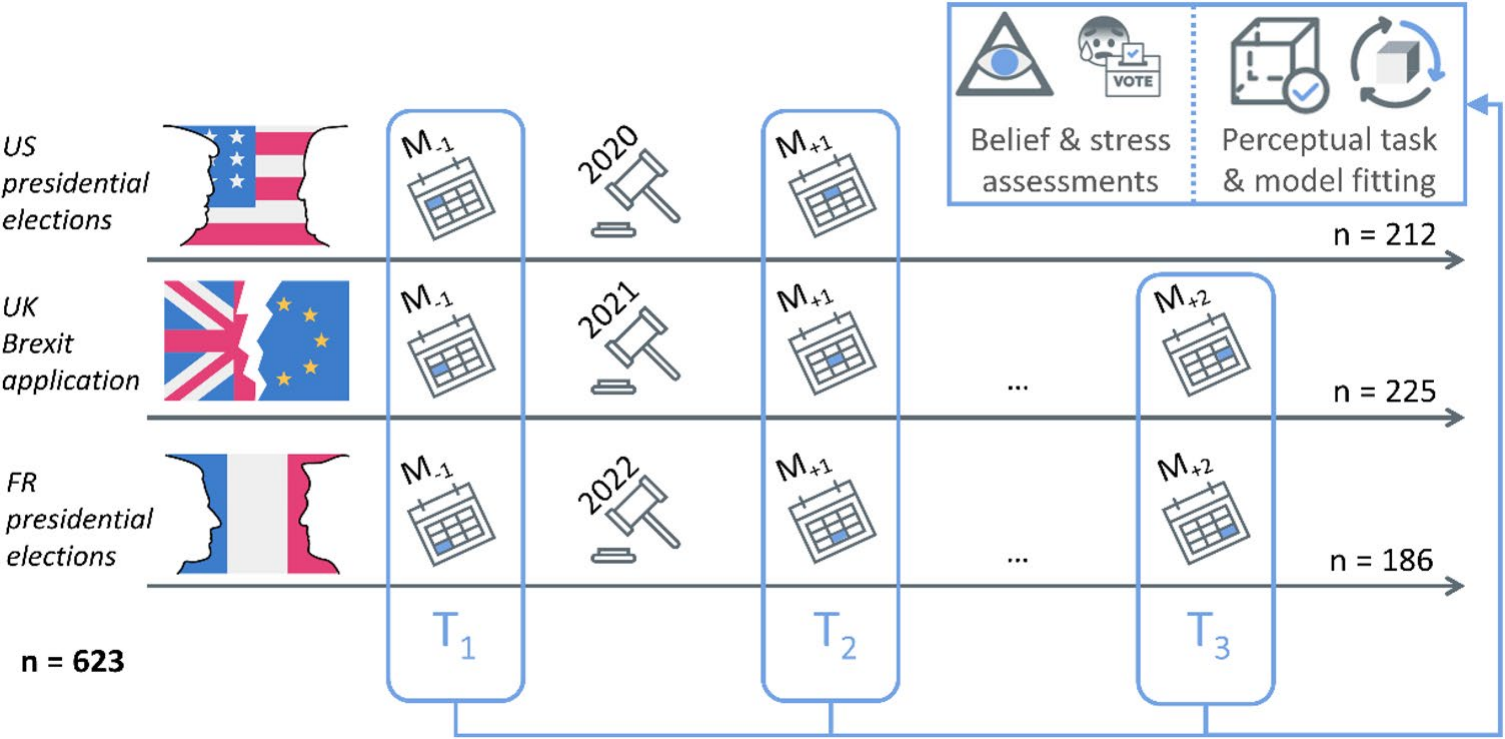
A repeated-measures design framing stressful political events in 3 different countries. Conspiracy ideations, political distress and perceptual stability were measured in the same participants (n = 623) via an online procedure, before and after the occurrence of a polarizing political event in three Western countries (M stands for month): the 2020 presidential election in the *United States of America* (n = 212, US), BREXIT implementation in the *United Kingdom* (n = 225, UK) and the 2022 presidential elections in *France* (n = 186, FR). We used T1 and T2 measures in the main model, while T3 was used in control analyses (see *Supplementary Material section: Controlling for experimental design biases*).

Because we assumed that periods of great sociopolitical uncertainty lead to significant increases in individual levels of distress and favor inferential biases such as conspiracy endorsements, we explored conspiratorial beliefs and perceptual stability around polarizing political events in three independent Western countries (see Fig. 1): the *United States of America* (US, 2020 presidential elections), the *United Kingdom* (UK, 2021 BREXIT implementation) and *France* (FR, 2022 presidential elections). At each time point, healthy participants were instructed to rate their level of distress related to the ongoing event in their own country (later referred to as *political distress*, see *Methods* and *Supplementary Material section: Self-reported measures*).

### Necker cube experiment

**Figure 2:**
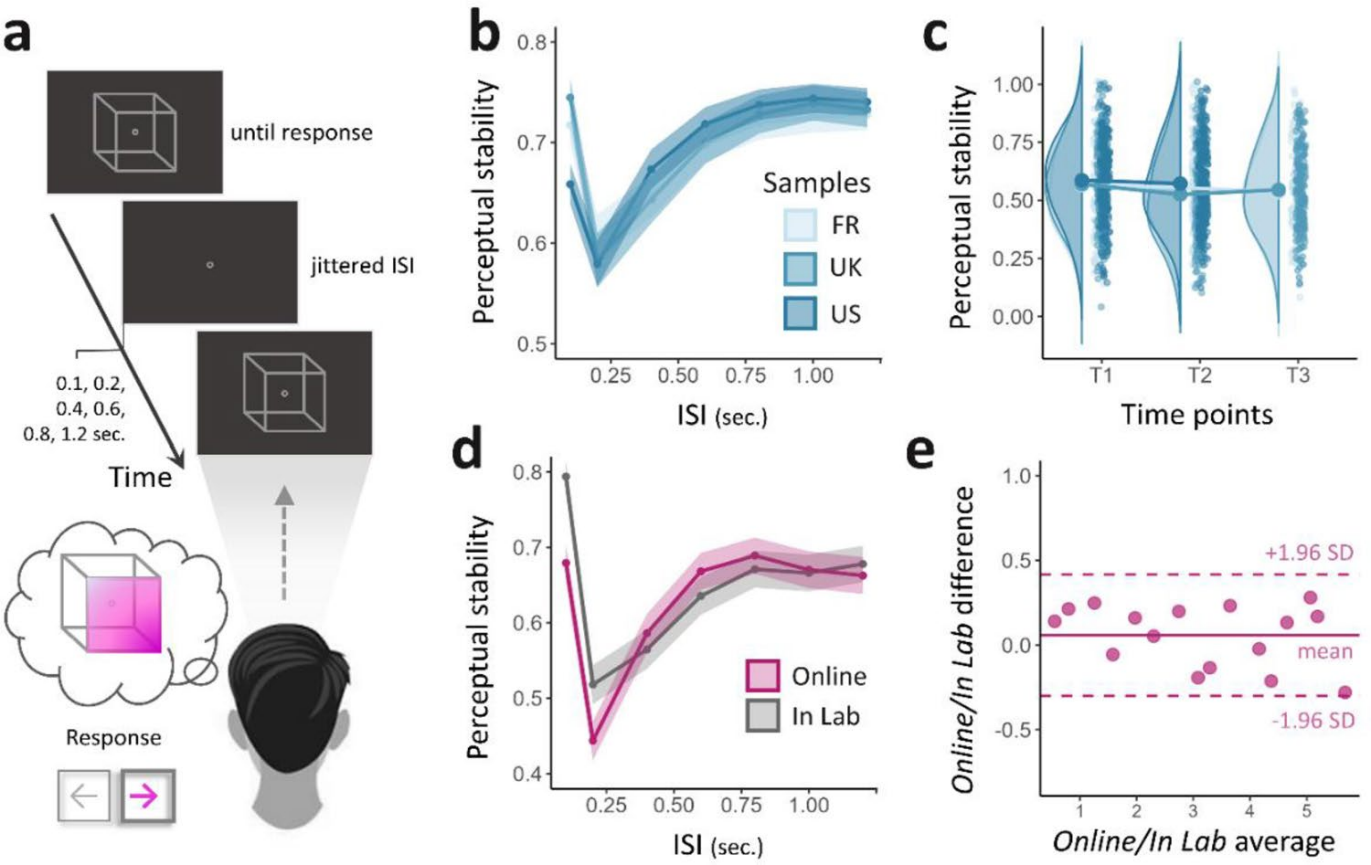
The Necker cube (NC) task: procedure and validity. (**a**) The experimental procedure consisted of serial NC presentations. Each trial was decomposed into three steps (see *Methods* section). After a fixation cross of pseudorandomized duration (ISI) (1), the Necker cube was presented (2) until participants reported their interpretation of the stimulus: ‘seen from above’ (SFA) or ‘seen from below’ (SFB), using the right or left arrow of their keyboard, respectively (3). (**b**) Perceptual stability as a function of the interstimulus interval (ISI) for each national sample. US (United States of America, mean Stability Score = .587, s.d. = .172), UK (United Kingdom, mean Stability Score = .570, s.d. = .176) and FR (France, mean Stability Score = .565, s.d. = .1472). (**c**) Averaged stability scores at each time point for the three national samples. (**d**) Perceptual stability as a function of ISI, for online (mean stability score = .441, s.d. = .190) and in-lab methods (mean stability score = .500, s.d. = .140). (**e**) Bland–Altman plot of the agreement between online and in-lab methods comparing stability scores obtained in each condition for the same participants (n = 16). The x-axis represents the average scores of the two methods. The y-axis represents the mean difference between online and in-lab stability scores. The limits of agreements (LoA, pink dotted lines) are defined as the mean difference computed on all participants (pink line) ± 1.96 s.d., and each dot represents a participant. As all participants are included in the LoA, the methods are considered to be in agreement and may be used interchangeably.

At each time point, the 623 enrolled participants performed an online bistable perception task based on the Necker cube (NC). The interpretation of the two-dimensional NC projected from a three-dimensional space naturally alternates between two possible configurations: a *seen from above* (SFA), or a *seen from below* (SFB) cube (Fig. 2a). A perceptual *stability score*, ranging from 0 to 1, was estimated at the participant level. This score corresponds to the probability of switching from one interpretation to the other (0 means total instability, while 1 reflects a perceptive rigidity where the participant only sees one interpretation of the two, see the *Methods* section). Assuming a universal mechanism at the roots of belief formation, we merged the 3 samples after ensuring their comparability in terms of perceptual stability at baseline (Table 1, Fig. 2b,c; see also *Supplementary Material section: Controlling for experimental design biases*). Importantly, perceptual stability was tested for *in lab/online* within-subject reproducibility on a pilot independent sample before running the final online experiment (Fig. 2d,e). We also ensured that dynamic changes in stability between the different time points were not due to a simple training effect between the sessions (see *Supplementary Material section: Controlling for experimental design biases*).

**Table 1:** Description of the populations at baseline.

	Sex (F /M)	Age (y.o.)	Education	pol. distress	GCB	stability
Whole Sample (n = 623)	310 / 311	33.0 ± 10.9	5.64 ± 1.41	5.07 ± 3.48	33.8 ± 13.3	.572 ± .178
US (n = 212)	119 / 106	30.4 ± 9.83	5.22 ± 1.43	4.60 ± 3.88	37.7 ± 13.9	.585 ± .173
UK (n = 225)	98 / 112	38.2 ± 10.7	5.52 ± 1.44	5.69 ± 3.32	33.3 ± 13.7	.566 ± .178
FR (n = 186)	93 / 93	29.8 ± 9.90	6.25 ± 1.12	4.87 ± 3.07	30.0 ± 11	.565 ± .183
Cross-country differences	*p* = .431	*p* < .001	*p* < .001	*p* < .01	*p* < .001	*p* = .442

US: United States of America; UK: United Kingdom; FR: France; F/M: female or male; y.o., years old; Education levels are provided according to the International Standard Classification of Education (ISCED); pol. distress: political distress; GCB: Generic Conspiracist Beliefs Scale; stability: fitted stability score (see *Methods section: Judgment criterion*); *p*: *p*-value. The sex-ratio did not differ across samples (X^2^ = 1.68, *p* = .431). UK participants were significantly older (F(2,408) = 44.255, *p* < 0.001, η^2^ = 1.29e^-19^) and demonstrated a higher level of political distress (F(2,408) = 5.8388, *p* < .01, η^2^ = 2.82e^-3^). FR participants reached a higher educational attainment (F(2,411) = 35.458, *p* < 0.001, η^2^ = 3.76e^-13^) than the other samples while US participants endorsed stronger conspiratorial beliefs (F(2,412) = 19.038, *p* < .001, η^2^ = 3.48e^-8^). Finally, stability was consistent across samples (F(2,405) = 0.81828, *p* = .442).

### Conspiracy adherence measures

**Figure 3:**
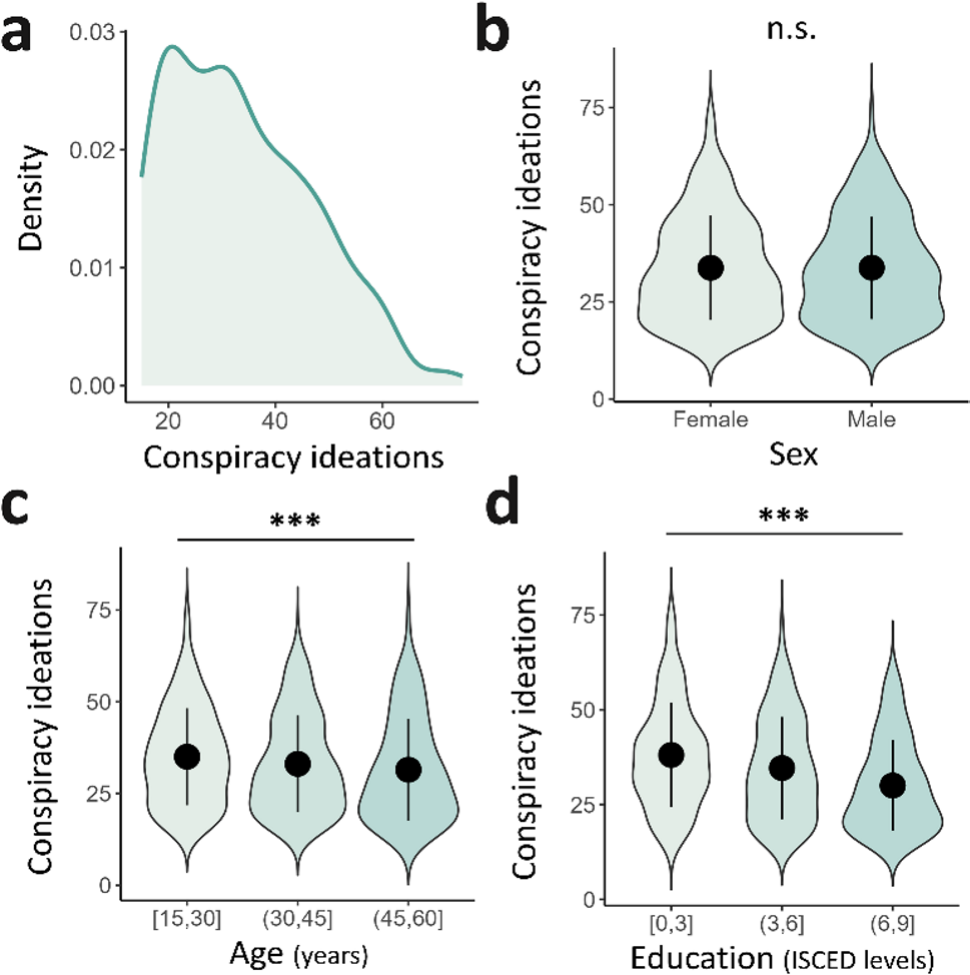
Sociodemographic features associated with conspiracy theories at baseline. (**a**) Left-skewed distribution of GCB scores across the entire international sample (N = 623). (**b**) Mean conspiracy scores in females (n = 310, mean = 33.8, s.d. = 13.5) and males (n = 312, mean = 33.8, s.d. = 13.2). The between groups difference was not significant. (**c**) Mean conspiracy scores according to age level. *Young* participants (n = 310, age = [18;30]) displayed higher GCB scores (mean = 35, s.d. = 13.2) than the *adults* (n = 210, age = (30;45], mean = 33.1, s.d. = 13.2) and older *adults* (n = 103, age = (45;60], mean = 31.5, s.d. = 13.8). (**d**) Mean conspiracy scores according to educational attainment levels. *The low education* group (n = 86, ISCED = [0;3]) scored significantly higher on GCB (mean = 38.2, s.d. = 13.7) than the *medium education* (n = 363, ISCED = [3;6], mean = 34.7, s.d. = 13.5) and the *high education* groups (n = 179, ISCED = [6;9], mean = 30, s.d. = 12).

The participants were instructed to self-rate their level of adherence to CTs, completing the *Generic Conspiracist Beliefs Scale* (GCB, see *Methods* section) at each time step. Replicating previous findings, we showed that conspiracy ideations were not normally distributed across the tested participants (W = 0.954 ; *p* = 0.440e^-12^, Fig. 3a, Fig. S1a), suggesting that only a subpart of the general population commonly endorses such beliefs. The distribution of the total GCB scores differed across the three samples (χ^2^ = 31.5, *p* < 0.001, η^2^ = 0.348e^-07^) despite a similar pattern across subscales (Fig. S1a-b, Table S1), notably demonstrating a common preoccupation for information control.

Looking more precisely at the sociodemographic features associated with conspiracy endorsement, we replicated previous findings from the literature (see *Supplementary Material section: Sociodemographic features of conspiracy theories*), notably showing that despite an absence of a link with the sex of participants (Fig. 3b), GCB scores significantly differed as a function of age (F(2,620) = 3.10, *p* = 0.046, η^2^ = 0.039, Fig. 3c), education (F(2,620) = 13.5, *p* < 0.001, η^2^ = 0.395e^-05^, Fig. 3d) and country (F(2,412) = 19.038, *p* < 0.001, η2 = 3.48e^-8^, Fig. S1a). Thus, we retained those variables as covariates for later analyses.

### Stress correlates at baseline

**Figure 4:**
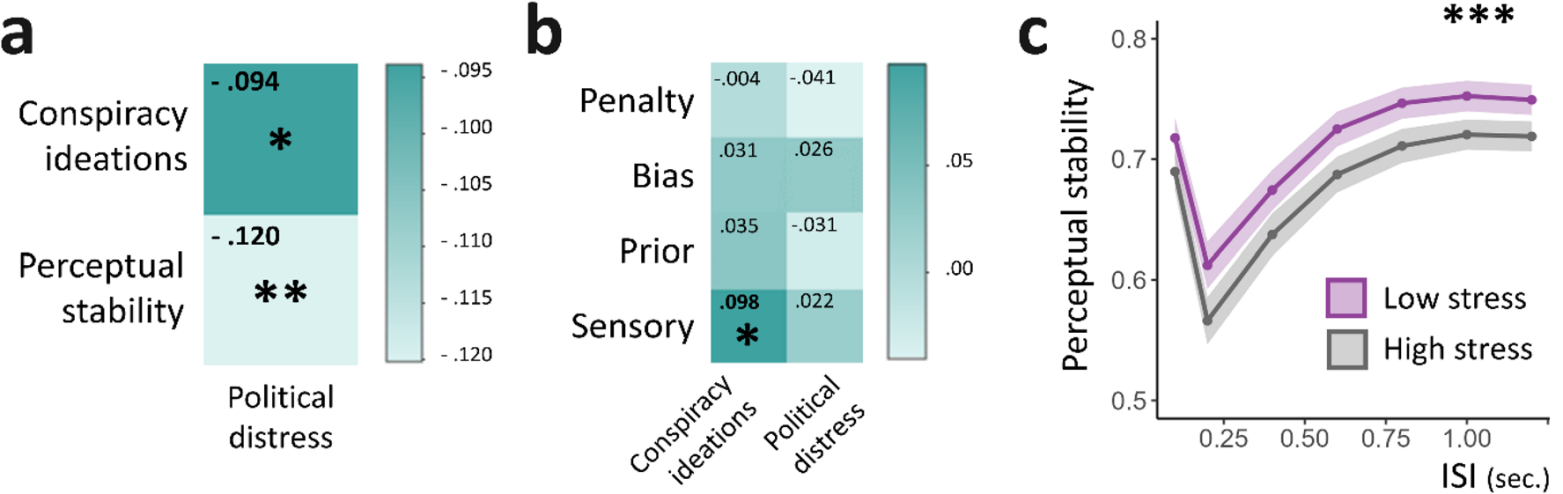
Cognitive and perceptual inference correlates at baseline. (**a**) Heatmap depicting the strength of associations at baseline between political distress, conspiracy ideations measured with the *Generic Conspiracist Beliefs Scale* (GCB) and perceptual stability (Spearman’s correlations, corrected for multiple comparisons using the *false discovery rate* method, FDR). Political distress was negatively associated with GCB (ρ =  − .094) and perceptual stability scores (ρ =  − .120). (**b**) Heatmap illustrating the strength of associations at baseline between GCB scores, political distress and Circular Inference parameters (sensory weight ($w$), prior amplification (${L}_{St}$), penalty term ($P$) and bias (${r}_{on}-{r}_{off}$). Spearman’s correlations were corrected for multiple comparisons using FDR. GCB scores were significantly associated with sensory overweighting (ρ = .098). (**c**) Perceptual stability plotted as a function of inter-stimulus-interval (ISI) in *low stress* (LS) group (n = 310, mean political distress = 2.10, s.d. = 2.06; mean stability score = .597; s.d. = .176) and *high stress* (HS) group (n = 313, mean political distress = 8.01, s.d. = 1.58; stability score = .548; s.d. = .177) groups. Perceptual processing was found significantly more rigid in the LS group than in the HS group (Cohen’s *d* = .140;). * stands for *p* < .05, ** for *p* < .01 and *** for *p* < .001.

We assume that some participants might adopt information-processing strategies that can reduce the uncertainty induced by the framed political event. Notably, we expect that the search for stability would translate into high levels of confidence measurable at different levels of processing, from perception to conspiracy beliefs. Since belief in CTs has been proposed to be a coping strategy able to reduce the stress elicited by uncertainty, we also expect an association between great levels of confidence and low levels of distress. We first checked for associations between political distress at baseline (i.e., when uncertainty peaked) and: (i) perceptual stability on the one hand, and (ii) conspiracy endorsement on the other hand (Fig. 4a). Political distress was found to be negatively linked with both levels of inference (*p* = 0.028, ρ =  − 0.120 and *p* = 0.007, ρ =  − 0.094 respectively). We further confirmed these findings by splitting the sample into two subsamples according to stress: (i) a 'low stress' (LS) and (ii) a 'high stress' group (HS). Comparing these two groups at baseline, we confirmed a significant difference in both stability (U = 41,385, *p* = 0.002, Cohen’s *d* = 0.140, Fig. 4c) and GCB scores (U = 43,411, *p* = 0.023, Cohen’s *d* = 0.110), such as the LS group scored higher in both.

Then, we examined the influence of age, education, country, political distress and perceptual stability on GCB scores (F(6,616) = 11.24, *p* < 0.001, adjusted R^2^ = 0.090). We found that age (estimate =  − 0.142, *p* = 0.006), education (estimate =  − 1.35, *p* < 0.001) and country (*p* < 0.001) were significantly associated with CTs, which further confirmed that political distress (estimate =  − 0.405, *p* = 0.008) is associated with conspiracy endorsement, even after we controlled for those sociodemographic factors.

### Fitting the circular inference model

Perception can be conceptualized as an inferential process in which noisy or ambiguous sensory data are combined with prior beliefs using the Bayes theorem^46–49^. However, humans are frequently utilizing suboptimal probabilistic reasoning. We previously suggested that deviations from “Bayes-optimality” could be caused by the circular inference (CI) phenomenon, i.e., internal amplification priors and sensory evidence through feedforward/feedback loops in brain circuits^50^.

To better understand the association between conspiracy theories and perceptual inference, we fitted a dynamical Circular Inference model to the Necker cube (NC) task^51^. When applied to this type of behavioral data, the CI model describes the process through which participants combine prior expectations about the visual appearance of three-dimensional (3D) objects and ambiguous visual input (such as illusory depth cues) to compute a 3D interpretation of the two-dimensional (2D) NC, as seen from above (SFA) or seen from below (SFB).

**Figure 5:**
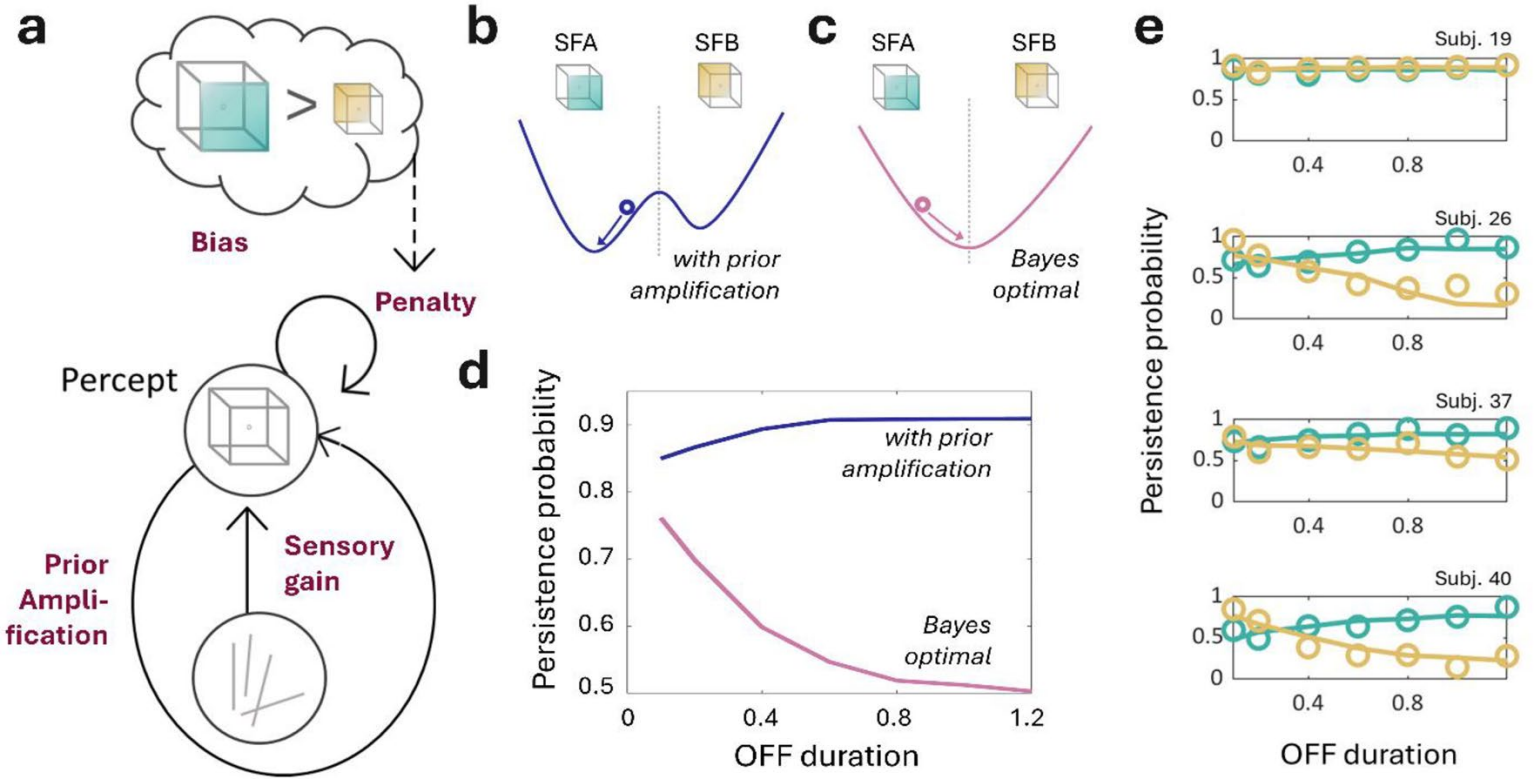
The circular inference model. (**a**) The Circular Inference (CI) model relies on 4 complementary parameters described as follows: (1) the overall *gain* based on the sensory inputs ($w$), (2) an *amplification of prior* beliefs due to the presence of descending loops (${L}_{St}$), (3) a ON/OFF switch penalty causing *volatility* in percept generation ($P$), and (4) the configuration preference, or *bias for the dominant percept* (${r}_{on}-{r}_{off}$) (see also *Methods*). (**b**,**c**) present the energy landscapes of the model, with and without prior amplification. (**b**) In the presence of noise, the prior amplification generates a bistable attractor allowing for a stabilization of the 3D configuration. SFA corresponds to the « seen from above » configuration, while SFB corresponds to the « seen from below » one. (**c**) On the contrary, a model without prior amplification (i.e., Bayes-optimal case) meets difficulties to stabilize on a given percept. (**d**) represents these patterns by plotting the persistence probability (PP) for both SFA and SFB. While the simple Bayes model exhibits a fall in the PP when OFF duration increases, the CI model allows for stabilized percept. (**e**) Illustration of the CI ability to capture the between-subject variability in the Necker cube bistable task. The cube PP is plotted as a function of the OFF duration for 4 random participants taken from the study. Circles correspond to experimental data and lines to the model fitting.

Figure 5 illustrates why a CI model may capture bistable perception more accurately than a Bayes-optimal model. Here, the two possible 3D configurations for the Necker cube (SFA or SFB) are represented as a single binary variable (Fig. 1a). The 3D configuration persists over time but with some volatilities, e.g., occasional switches from SFA to SFB, and vice versa. Low-level sensory features (contours, disparity, etc.) may support either SFA (positive sensory input) or SFB (negative sensory input). When the Necker cube was ambiguous, we modeled the sensory input as white noise, with a mean of zero and unit variance representing sensory noise.

We represent the subject's internal belief as a value that is positive or negative if SFA or SFB, respectively, is perceived with high confidence but near zero in the case of uncertainty (see *Methods*). In a Bayes-optimal model, the percept corresponds to a leaky integration of sensory inputs over time, and sensory gain and leak are determined by sensory precision and volatilities. This process can be conceptualized as sensory noise pushing around a ball situated in a bowl-shaped energy landscape (Fig. 5b). The percept (or most likely configuration) corresponds to SFA or SFB when the ball is situated on the right or left side of the bowl, respectively. The higher parts of this landscape correspond to high levels of confidence in the current percept, while the lower parts correspond to higher levels of uncertainty. Notably, because of volatility, the ball will spend more time in this region of uncertainty. Moreover, in the absence of sensory inputs to push it around, the ball will eventually fall to the bottom of the bowl (equiprobability of the SFA and SFB configurations). In other words, each percept loses its influence over time, and the probability of persistence decreases continuously in the absence of sensory input (which corresponds to the OFF durations of our NC behavioral task; Fig. 5d, dark yellow line). This first approach appears to contradict experimental observations (replicated in this study) that bistable perception is stabilized for longer “OFF” durations^52^.

In contrast, in the presence of CI, sensory inputs are pushed in the direction of prior beliefs and influence future beliefs in turn. This will result in an amplification of the prior at the detriment of sensory evidence (Fig. 5a, blue arrow). Thus, the energy landscape becomes bimodal with two valleys that correspond to the SFA and SFB configurations (Fig. 5b). As a result, CI consistently generates stable and strong beliefs (the ball remains in the same valley for long periods), with sensory noise occasionally causing a perceptual switch (making the ball fall in the opposite valley). In the absence of sensory input, the ball falls to the bottom of the valley in which it is currently located and where it remains stuck. Thus, the influence of the previous percept does not decay over time, and perceptual switches become less likely to occur for longer OFF durations (Fig. 5d, green line), in better agreement with what has been previously observed at the behavioral level. Finally, SFA is perceived more frequently by most people and can be captured by different switching frequencies (volatilities) between SFA and SFB. This bias renders the SFA valley deeper and the “SFB” valley shallower and predicts a less stable or even an unstable SFB percept, as indeed observed in many subjects (see, for example, the second and fourth subjects from the top in Fig. 5e).

By using this approach, we could fit four model parameters contributing to the perceptual decision of each individual subject: the weight of the sensory gain (sensory), the amplification or prior beliefs (prior), the strength of the bias (bias), and a fourth parameter (penalty). This fourth parameter can be conceptualized as the reflection of an adaptive strategy that enables the perception of the less probable configuration to occur at least some of the time (see *Methods*). By using this model, we were able to capture a wide variety of responses (Fig. 5e). We checked whether these CI parameters could capture the effects of political distress and conspiracy adherence. Sensory weight was the only parameter positively associated with GCB scores at baseline (*p* = 0.030, ρ = 0.098, Fig. 4b), supporting the idea that participants more prone to CTs at baseline rely more on sensory evidence when asked to make a decision in a highly ambiguous environment. We confirmed this GCB-sensory weight association (estimate = 1.20, *p* = 0.051) even after controlling for the effects of age, education and political distress (F(4,618) = 11.86, *p* < 0.001, adjusted R^2^ = 0.065).

### Measured changes after political event resolution

**Figure 6:**
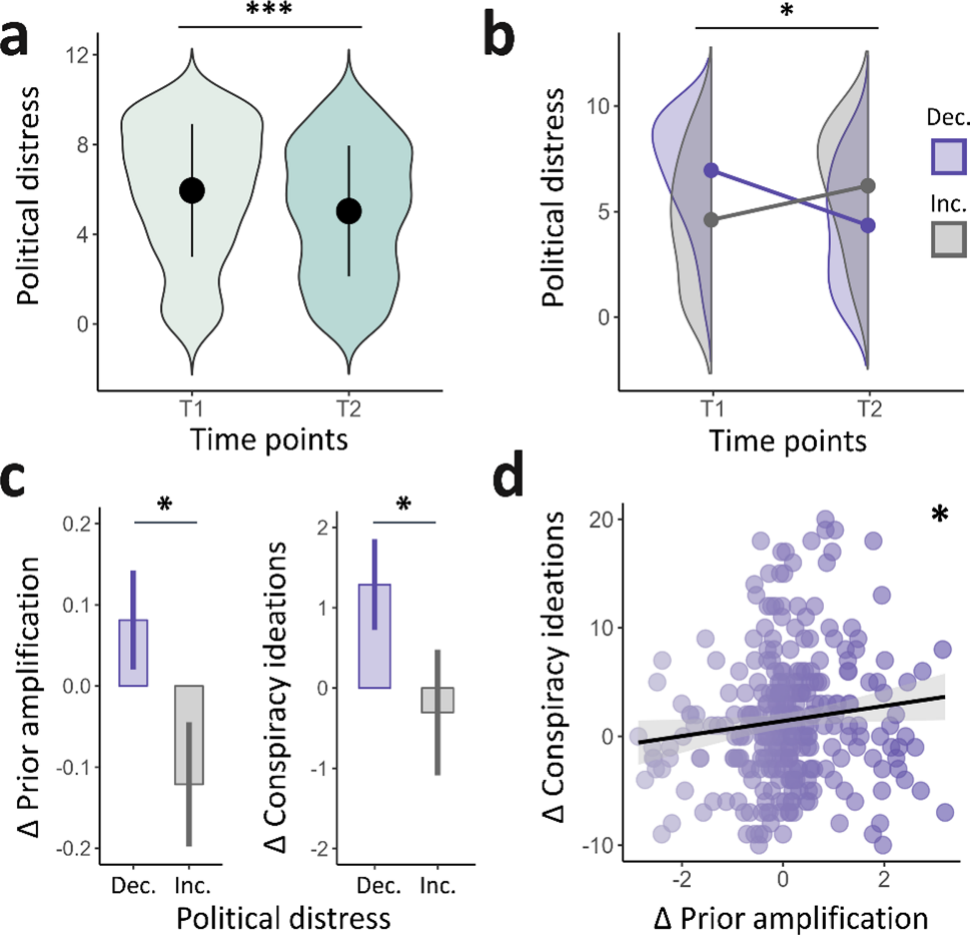
Computational and cognitive features associated with changes in political distress over time. (**a**) Illustration of political distress scores over testing sessions. Political distress decreased between baseline (T1, mean political distress = 5.35, s.d. = 3.33) and retest (T2, mean political distress = 4.54, s.d. = 3.16; Cohen's *d* =  − .250). (**b**) Political distress over time according to stress trajectories. Some participants showed decreased political distress after uncertainty resolution (Dec, n = 330, mean Δpolitical distress =  − 2.78, s.d. = 2.45), while another subsample showed increased stress (Inc, n = 220, mean Δpolitical distress = 2.01, s.d. = 2.14). (**c**) Left: Illustration of the evolution of prior amplification (ΔPrior) over time for the ‘Dec’ (mean = .081, s.d. = 1.11) and ‘Inc’ (mean =  − .121, s.d. = 1.14) groups. The intergroup difference was significant (Cohen’s *d* = .180). Right: Evolution of conspiracy ideations (ΔConspiracy ideations) over time for the ‘Dec’ (mean = 1.29, s.d. = 10.3) and ‘Inc’ (mean =  − .305, s.d. = 11.6) groups. The intergroup difference was significant (Cohen’s *d* = .145) (**d**) Scatter plot showing the correlation between ΔPrior and ΔConspiracy ideations in the ‘Dec’ group (*p* = .035, ρ = .116; Spearman correlation, ρ = .116). * indicates *p* < .05, *** indicates *p* < .001.

We then assessed changes in political distress, conspiracy ideations and perceptual stability over time (Table 2). We confirmed an overall stress reduction at T2 compared to that at baseline (W = 100,834, *p* < 0.001, Cohen's *d* =  − 0.250 ; Fig. 6a), despite some heterogeneity in the participants. Meanwhile, GCB scores significantly increased (W = 73,048, *p* = 0.017, Cohen's *d* = 0.068), while stability scores decreased (W = 114,427, *p* < 0.001, Cohen's *d* =  − 0.139)—this tendency toward destabilization was observed in each national sample (see also Fig. S2).

**Table 2:** Population description at each time-step: scores, and CI parameters.

	Pol. distress	GCB	Stability	Sensory	Prior	Bias	Penalty
T1	5.35 ± 3.33	33.78 ± 13.33	.57 ± .18	1.70 ± .85	1.85 ± .91	.59 ± .07	− 2.04 ± 1.50
T2	4.54 ± 3.16	34.70 ± 13.56	.55 ± .18	1.65 ± .85	1.86 ± .93	.59 ± .06	− 2.20 ± 1.40

Pol. distress: political distress; GCB: Generic Conspiracist Beliefs Scale; stability: estimated stability score (see also *Methods section: Judgment criterion*); Sensory: sensory overweighting ($w$); Prior: prior amplification ($a$).

To account for the heterogeneity in stress evolution, we split the sample into two subgroups according to their trajectories: a first subsample with decreasing stress (Dec, n = 330) and a second subsample with increased stress between T1 and T2 (Inc, n = 227; Fig. 6b). Considering that the Dec group should have adopted the most efficient coping strategies, we checked how the CI parameters and degree of conspiracy ideations changed over the same period in these two subsamples (Table S3).

A delta measure for each CI parameter was computed (parameter value at retest minus value at baseline), such as a positive delta indicated a gain in the parameter value, while a negative delta reflected a decrease in this parameter. The Dec group showed increased reliance on prior information in the bistable task between T1 and T2 (mean ΔPrior = 0.0811, s.d. = 1.11), while the Inc group showed decreased use of priors in the same period (mean ΔPrior = -0.121, s.d. = 1.14). This difference was statistically significant (t(460,65) = 2.07, *p* = 0.039, Cohen’s *d* = 0.180 ; Fig. 6c-left). We found no differences in the 3 other CI parameters (Fig. S5).

We also computed a composite ΔGCB score corresponding to GCB at retest minus GCB at baseline, such that a positive delta corresponded to an increase in conspiracy adherence, while a negative delta resulted in a decrease. Because conspiracy ideations were proposed to act as a coping mechanism when facing uncertainty, we next ran an oriented test to test this hypothesis, which confirmed conspiracy strengthening among participants with decreased stress in comparison with that observed in the rest of the sample (t(429,52) = 1.65, *p* = 0.050, Cohen’s *d* = 0.145; Fig. 6c-right). This finding supports a gain in the GCB score for the Dec group compared to the Inc group. To confirm that the increase in GCB score was directly associated with an increase in Prior in the Dec group, we compared ΔPrior and ΔGCB in this specific subsample; these measures were found to be positively associated (*p* = 0.035, ρ = 0.116, Fig. 6d).

## Discussion

A surge in CTs has been observed in recent years, and CTs have been proposed to act as coping strategies for the stress and perceived lack of control generated by global uncertainty^14–18^. CTs offer intuitive and easy-to-understand explanations to unsolved problems^53^. Links have already been established between conspiracy endorsement and some inference biases^6,19–21^. However, very few studies have primarily focused on low-level perceptual aspects of conspiracy^27,28,31,33^, and limited efforts have been made to delve into the potential mechanisms of information processing that may convey such associations.

To address these concerns, we combined online assessments of bistable perception in large international samples with Bayesian modeling. By using this approach, we could quantify perceptual inference mechanisms and test their links with conspiracy ideations during periods of great sociopolitical uncertainty. We were able to capture the strengthening of conspiracy beliefs in nonclinical populations. Specifically, using the Circular Inference (CI) model, we highlighted a significant association between conspiracy endorsement and the overweighting of sensory information in the wake of political polarizing events, which was followed by a selective increase in prior reliance in those who subsequently decreased their stress levels.

Several attempts to model the features of conspiracy beliefs can be found in the literature. However, most of these models have either focused on the network scale^54^ or remained purely theoretical, without experimental testing^40^. Recent findings have highlighted the added value of a computational framework to account for the emergence of conspiratorial beliefs during the COVID-19 pandemic^35^ and the protective aspect of CTs against distress in a social context^55^. These studies used high-level cognitive tasks and focused mainly on paranoia, a condition that shares some phenomenological features with CTs but is also considered to be significantly different^56^, further justifying specific explorations. The quantitative approach proposed in the present work nicely completes these initiatives, adding to the testing of low-level inference, together with measurements of the emergence and strengthening of conspiracy beliefs.

In this study, we provide the first evidence for an association between sensory information overweighting in ambiguous contexts and a high level of conspiracy endorsement. This finding suggests that when uncertainty is assumed to peak, a subpart of the population that is more vulnerable to stress is prone to embracing conspiracy explanations based on intuitive reasoning. Motivated by the need to cope with uncertainty, these participants first adopt an “exploration” strategy, seeking explanations in their direct environment to inform their perceptual decisions. Interestingly, such a mechanism accounts for perceptual and inferential biases previously found to be associated with conspiracy ideations, such as illusory pattern detection^27,28,33^, aberrant salience attribution^22^, intuitive thinking^57,58^ and the JTC phenomenon^23,24^.

We also explored the dynamic changes in model parameters after stress resolution by using a pre/post design surrounding the political events. We shed light on the association between prior knowledge amplification in perceptual decisions and the enhanced adherence to CTs in those who showed reduced stress level. This finding suggests that some participants coped with uncertainty by embracing conspiracy-oriented explanations, secondarily shifting to an “exploitation” strategy (Fig. S6), validating their newly established view and reinforcing their own beliefs. This second mode appears compatible with findings showing confirmation biases^59,60^ and reality testing deficits^25^ in people with CTs, making these beliefs more resilient to counterevidence.

These results can also be compared with models of the emergence and maintenance of clinical beliefs, such as delusional ideations. Indeed, prior research conceptualized delusion formation as the result of impaired associative learning processes driven by excessive prediction error^61^, a framework that was later extended to account for delusion persistence as aberrant reinforcement of previously learned associations^62^. Our results also add to previous work showing that parametric changes might mimic behaviors observed during the transition to psychosis^63^. It was shown using CI-based simulations that the seminal amplification of sensory information involved in the integration of aberrant causal relationships (during the transition to psychosis) subsequently constituted strong priors proposed as responsible for the stability of delusional contents from one psychotic episode to the next. Both approaches (predictive coding and Bayesian modeling) are congruent with (i) the idea that conspiracy endorsement is associated with the establishment of aberrant causal relations between random events^14^, and (ii) that conspiracy could be rooted in the self-reinforcement of previously integrated suboptimal beliefs.

While endorsing CTs may serve as an effective short-term coping strategy, it also appears to pave the way for the long-term strengthening of suboptimal beliefs (beliefs that would be computed through mechanisms deviating from Bayes’ rule), making it maladaptive for stress regulation overall. Gaining a better understanding of this phenomenon has vast social implications. Humankind has experienced repeated periods of heightened uncertainty throughout history, ranging from civilizational collapses or wars to economic crises. In extending the well-established association between political distress and the endorsement of CTs^13^, our model also explains the recent rise in extremism and populism observed since the beginning of the twenty-first century in the global context of the pandemic, terror attacks and climate change.

We must acknowledge that this work has some limitations. First, although significant, some results exhibit small effect sizes (i.e., Cohen’s *d* of approximately 0.2) and are not always replicated when countries are tested separately. Of note, small effect sizes were previously found to still have substantial significance when studies were conducted on large populations^64^. Importantly, small effects were expected because we attempted to capture an association between a low-level inference process (bistable perception) and a more complex cognitive process (conspiracy). However, these findings still constitute an important proof-of-concept demonstration that the CI model can capture small variations in nonclinical populations’ perceptual decisions, paving the way for promising advancements in deepening our understanding of the mechanisms underlying belief rigidification.

A second limitation is that we cannot rule out that some participants may have felt hesitant in honestly reporting their views about CTs, due to the controversy and potential stigma surrounding conspiracy thinking. However, we think that our experimental design offers two advantages in the valid assessment of conspiracy endorsement. First, its online nature ensured anonymity and encouraged freedom of speech, as frequently observed on the internet and digital social media. Second, the joint use of a low-level perceptual task, the NC, provided access to a proxy of inference processing that is rarely prone to social biases, such as interviewer compliance.

A third limitation is the representativity of the sample: we indeed chose to recruit participants from three Western educated countries that are known for their high degree of polarization^65^. Although our sample may not represent the world population and various sociocultural factors can influence conspiracy adherence, we argue that the phenomenon under investigation follows some universal rules. First, links between sociopolitical uncertainty and the resurgence of conspiracy beliefs have already been observed at various times and locations, dating back to the Roman Empire^66^. Second, while the GCB total scores were distributed differently among our three samples (Fig. S1a), their qualitative distribution across GCB subscales followed the same pattern (Fig. S1b). Third, the pattern of associations between political distress and inference processing measures (perceptual stability and CI parameters) appears to be consistent across the three samples when tested separately (Fig. S2).

For the same reasons, we focused on the level of distress related to specific political events in the countries where we conducted the tests. Importantly, we did not consider other types of individual stress levels. Instead, we concentrated on the broader phenomenon of sociopolitical uncertainty. Similarly, our procedure did not allow us to have a direct measure of this uncertainty, which could constitute an interesting addition in future studies. Finally, while we observe an increase in GCB scores in some subpopulations, this phenomenon, referred to as “CTs strengthening,” could be due to (i) a widening range of CTs rated as believable across time (scoring on more items of the scale) or (ii) a strengthening of conviction (scoring higher on the same items).

Overall, this study highlights the potential of the Circular Inference model in examining subtle variations in inference processing associated with high-level cognitive beliefs. This model has already proven effective in accounting for the positive symptoms of schizophrenia^43,44,67^ and schizotypal traits^46^; however, this breakthrough opens up new avenues for applying quantitative approaches to dynamically explore subjective beliefs in nonclinical populations. By applying this computational framework, we delved deeper into the mechanisms underlying the emergence and maintenance of conspiracy beliefs, shedding light on their societal impact and providing insights that could be valuable for developing interventions aimed to counter the influence of CTs during highly uncertain periods.

## Methods

### Participants

Three independent samples were recruited using the Prolific^©^ web-platform: 212 US citizens, 225 British citizens and 186 French citizens. The same protocol was administered 1 month before and 1 month after a major stressful political event: the 2020 US presidential election, the 2021 UK BREXIT implementation and the 2022 French presidential election (Fig. 1). The targeted participants were aged between 18 and 60 and had normal-to-corrected vision. They were from the nationality of the country of interest for each sample and regularly used social media. The exclusion criteria were a history of psychiatric or neurological disorder, strabismus, or eye surgery. From the initial sample (N = 755), 30 participants were excluded based on failed attentional checks (see *Supplementary Material section: Controlling for experimental biases*) or low reaction times (mean reaction time < 300 ms), while 102 were lost longitudinally.

The Prolific^©^ web-platform (https://www.prolific.co/) ensures data privacy following standards of the European and UK data protection law (i.e., General Data Protection Regulation (GDPR), transposed into UK law as the UK GDPR). Informed consent was obtained from all participants and their sociodemographic characteristics were associated with their respective behavioural data through an anonymous ID randomly assigned at enrollment. This online study was approved by the ethics committee *Comité de Protection des Personnes Nord-Ouest IV*, and its methods complied with French regulations and were carried out in accordance with relevant guidelines.

### Apparatus

The protocol was implemented in PsychoPy v.3, exported and hosted online on the Pavlovia.org platform. For the perceptual part of the experiment, participants were instructed to stand in total darkness, approximately 60 cm away from the screen and adjust it to be perpendicular to the floor with their eyes aligned to the fixation cross displayed at the centre of the screen. The NC task and the self-reported assessment of beliefs were administered in a randomized order (see also *Supplementary Material section: Controlling for experimental biases*).

### The Necker Cube Task

#### Stimuli

Visual stimuli representing Necker cubes (NC) were displayed in the centre of a black screen. The stimulus size was standardized across the participants using a matching method based on a standard credit card displayed on the screen that the participant was required to adjust in size before starting the experiment (See demo available at: https://github.com/RenaudJA/Necker_cube_demo).

#### Procedure

The block-design of the task was inspired by Mamassian and Goutcher's^68^ protocol. During each block, a NC was presented discontinuously. Referring to a forced-choice methodology, we asked participants to report their interpretation of the stimulus using their keyboard each time a new cube appeared on the screen. The cube disappeared after a pseudorandom duration (**ISI** ranging from 0.1 to 1.2 s). Each recorded response constituted a trial, and the experiment was divided into 10 blocks of 64 consecutive trials (i.e., 640 NC presentations per run), providing a discontinuous sample of the participant's perceptual dynamics. A 10-s black screen display separated each block to minimize the influence of the previous block on later responses (Fig. 2a).

Participants were instructed to stare at the target located in the middle of the screen to neutralize the potential effects of eye movements. The two possible interpretations of the NC (SFA, SFB) were explicitly mentioned, and subjects were asked to look at the cube passively, without attempting to orient or force their perception. A short training session was performed beforehand to give participants the opportunity to become familiar with the stimulus and the task while ensuring that the instructions were well understood.

#### Judgment criterion

Various parameters can be used to understand and describe the phenomenon of bistable perception. We chose to focus on perceptual stability because we were interested in its dynamical dimension, i.e., how the system could stabilise and destabilise.

Perceptual stability is defined as the probability that a percept persists from one trial to the next. According to Markovian modeling, the current percept (one of the two interpretations SFA or SFB) depends on the previous percept and its updating by sensory observation. This implies a circularity in the integration of information where the percept at time *t* becomes the prior information at time *t* + *1*. A value was thus assigned to each trial "i": 0 if the response was different from the response to trial "i-1" and 1 if the response to trial "i" was identical to the response to trial "i-1". The average SP was thus calculated for all trials and separately for each interpretation (SP0 and SP1 for SFA and SFB, respectively). Overall, the SP was interpreted as the general probability that the system remains stable from one trial to the next, where 1 corresponds to a system with no perceptual change and 0 to a system governed by maximum instability.

A previously proposed way to assess perceptual stability is by computing stability curves representing SP as a function of different ISI values. Such a curve usually consists of an initial “destabilization” portion corresponding to a drastic drop in perceptual stability, and a “stabilization” portion reaching a “ceiling threshold,” considered a good proxy of perceptual stability (Fig. 2b,d). This second portion of the curve was fitted to a reversed exponential function, and we considered the parameter corresponding to the last point of the curve as the stability score for each participant.

#### Self-reported measures

A sociodemographic form and some psychometric assessments were then conducted/collected on the Prolific^©^ platform. Participants specified their age and educational attainment as defined in the *International Standard Classification of Education* (ISCED)^69^. The participant demographics are shown in Table 1. When Likert or visual analogical scales were used, the cursor was coded to return to the centre of the screen after each question to avoid the answer being biased by previous ones. Adherence to CTs was assessed using the 15-item *Generic Conspiracist Beliefs Scale* (GCB)^70^ and its French translation^71^. The GCB scores and subscores for each sample are shown in Table 1 and Table S1. Participants were also asked to rate with a 10-point visual analogical scale how distressed they were regarding the target event in their country (political distress). The precise questions used are shown in the *Supplementary Material section: Self-reported measures*.

### The circular inference model

#### Dynamical equation

Belief updating in CI can be formalized as follows (see Leptourgos and colleagues^51^ for more detailed mathematical derivation):

1 $$\frac{dL}{dt}={r}_{on}(1+{e}^{-L})-{r}_{off}(1+{e}^{L})+aL+wS$$

With ${r}_{on}$ and ${r}_{off}$ corresponding respectively to the rate of switches from SFB to SFA and vice-versa; $L$ being to the log odd ratio of SFA versus SFB, $a$ controlling the strength of prior amplification, and $w$ being the sensory gain. Note that in the *Bayes-optimal* case, $a$ would be equal to 0.

We adapted the model (initially designed for continuous stimulus presentation) to intermittent stimulus presentation as follows. The input $S$ was assumed to be zero during OFF-periods, during which the belief evolved according to Eq. (1) with $wS$ = 0. At the onset of an ON-period, $S$ instantaneously increased or decreased $L$ by an amount first sampled from a normal distribution, then multiplied by the sensory gain (a noisy sensory input associated with the new stimulus). The model responds to the new stimulus according to the sign of $L$ following this update (i.e. SFA if $L$ > 0 or SFB if $L$ < 0).

On its own, we found that this dynamical equation could not fully account for responses to the 2 shortest OFF-durations in certain subjects. More specifically, subjects with particularly strong biases favoured “SFB” for the shortest OFF-durations, while strongly favouring “SFA” for longer durations (see, for example, Fig. 5e). This behaviour could be an adaptive strategy allowing for the perception of the less probable configuration to occur at least some of the time, as was instructed (otherwise, strongly biased subjects would be forced to respond “SFA” all the time). It could also be due to lower level sensory or response adaptation processes. To capture this effect without multiplying the number of free parameters, we used a free parameter $P$ that could be interpreted as an additional sensory input, induced by stimulus disappearance. Of note, while $P$ allowed for far better fits (leading to higher stability and confidence in the other fitted parameters), it was also strongly anti-correlated with the subject’s biases. As a consequence, after the model responds, $L$ is reset by a fixed amount that corresponds to this penalty term $P$ and a new OFF-period starts.

To predict individual responses, the model was tested on the same sequence of “OFF” duration that were used in each subject. We computed the pairwise statistics of two successive model responses (e.g. Probability of two successive SFA responses, SFA followed by SFB, SFB followed by a SFA, and two successive SFB, at each of the 7 delays, generating 28 measures). We then averaged the responses of 40 runs of the model (with identical OFF durations but different sensory noise samples). These predictions were compared with the pairwise statistics measured experimentally.

The CI model presented above could have up to 5 free parameters (${r}_{on}$, ${r}_{off}$, $w$, $a$, and $P$), while the *Bayes-optimal* model would have a maximum of 4 free parameters ($a$ being fixed to 0). We reduced the number of free parameters in the CI model in the following way. Let us define a bias $b$ (preference for SFA), such that ${r}_{on}=rb$ and ${r}_{off}=r\left(1-b\right)$, where $r$ is the mean volatility. The temporal dynamics of the model are dominated by its effective loop strength, ${L}_{St}=\frac{a}{r}$. If ${L}_{St}$< 1, the model acts as a leaky integrator, with “uncertainty” being the only stable state (Fig. 5c). On the other hand, if ${L}_{St}$> 1, the model becomes bistable for moderate biases (Fig. 5b), while only SFA is stable for stronger biases (i.e., the “SFB-valley" becomes too shallow to trap the ball). While ${L}_{St}$ has a crucial influence on perceptual choice dynamics, $r$ exerts only a moderate effect. Thus, we reduced the number of parameters by fixing it to 10 Hz for all subjects, corresponding to a mean sensory integration time constant of 100 ms. Thus, the 4 parameters of the CI model were the bias $b$, sensory gain $w$, loop strength for prior information ${L}_{St}$ and penalty term $P$.

While model comparison was not the main purpose of this study, we also tested a Bayes-optimal model (e.g., with $a$ = 0) with the same degree of freedom using the same methods, keeping b, w, r and P as its four free parameters. Indeed, without prior amplification, persistence following a long OFF duration is achieved only for very low volatilities ($\left(a*r\right)<\frac{1}{OFF-duration}$). We tested this model on 220 subjects extracted from the preelection American dataset. Our findings reveal a notable distinction in its ability to account for individual responses, as evidenced by the *mean squared error* (MSE) metrics (mean(MSE_ci_) = 0.075, mean(MSE_bayes_) = 0.093). This difference was statistically significant according to the Wilcoxon test, *p* = *2.673e*^-2^. Furthermore, the Bayesian information criterion (BIC) values corroborate these results, with BIC_ci_ =  − 549 and BIC_bayes_ =  − 502.

#### Model fitting procedure

For each model, we used MATLAB patternsearch to minimize the Euclidian distance between the predicted and measured pairwise response statistics for each OFF duration. For each subject and each session, patternsearch was repeated 100 times with different starting points, and the best parameter set was retained as the best model fit. We also performed parameter recovery and measured test–retest consistency between parameters measured in the same subject using data from the T2 and T3 time points (see *Supplementary Materials* and Fig. S4).

#### Data analysis and statistics

##### Characteristics of conspiracy adherence

The normality of the distributions was tested using the Shapiro‒Wilk test. If the data were not normally distributed, further analyses were performed using nonparametric statistics. We compared GCB scores between males and females using a Mann‒Whitney test, while GCB scores among the three US–UK–FR samples, across ISCED levels of education and across different age groups were compared using Welch ANOVAs.

##### The correlates of stress at baseline

We conducted a series of model-free analyses to confirm the association between political distress, stability score, and GCB. Again, due to the non-normal distribution of the GCB scores, we referred to Spearman rank correlations to explore linear associations, corrected for multiple comparisons based on the *false discovery rate* (FDR) method. These analyses were conducted on the whole sample, and on subsamples generated through a median split on the political distress score: the 'low stress' (LS, n = 310) and 'high stress' (HS, n = 313) subgroups. We used Mann–Whitney tests to assess the difference between these two subgroups regarding stability scores or GCB scores. We also used a linear regression model to confirm the association between political distress and GCB, adding age, education level and country as covariates to control for the effect of these sociodemographic factors.

##### Changes after political event resolution

We assessed the evolution of political distress, stability scores and GCB scores over time using Wilcoxon signed-rank tests for repeated measures. We then split our sample into two groups: Dec and Inc comprising individuals who showed decreased or increased stress, respectively, between the two time points. We computed a delta measure for each parameter that corresponded to the parameter’s value at retest minus that at baseline. A positive value indicated a gain in the parameter, while a negative value indicated a decrease. Due to the normal shape of distributions in these composite scores and our sample size, we referred to Welch tests for group comparisons.

The same procedure was used to compare the two groups regarding the gain in GCB (ΔGCB). We performed a Welch's test for the oriented hypothesis that the Dec subsample would significantly increase its GCB score compared with the Inc subsample. Finally, a Spearman correlation test was used to check for an association between ΔAlpha and ΔGCB in the Dec subgroup.

### Supplementary Information

Supplementary Information.

## Data Availability

The datasets used and/or analyzed during the current study are available from the corresponding author on reasonable request. A PsychoPy version of the task is available on GitHub: https://github.com/RenaudJA/Necker_cube_demo
