# Supplementary material for: Conspiracy beliefs and perceptual inference in times of political uncertainty
Source: Sci Rep. 2024 Apr 18;14:9001. doi: 10.1038/s41598-024-59434-4 (PMC11026417; doi:10.1038/s41598-024-59434-4)
Supplement: Supplementary file 1 — Supplementary Information. [file 41598_2024_59434_MOESM1_ESM.docx]

# **SUPPLEMENTARY MATERIAL FOR Salomé LECLERCQ et al. CONSPIRACY BELIEFS AND PERCEPTUAL INFERENCE IN TIMES OF POLITICAL UNCERTAINTY**

## Sociodemographic features of conspiracy theories


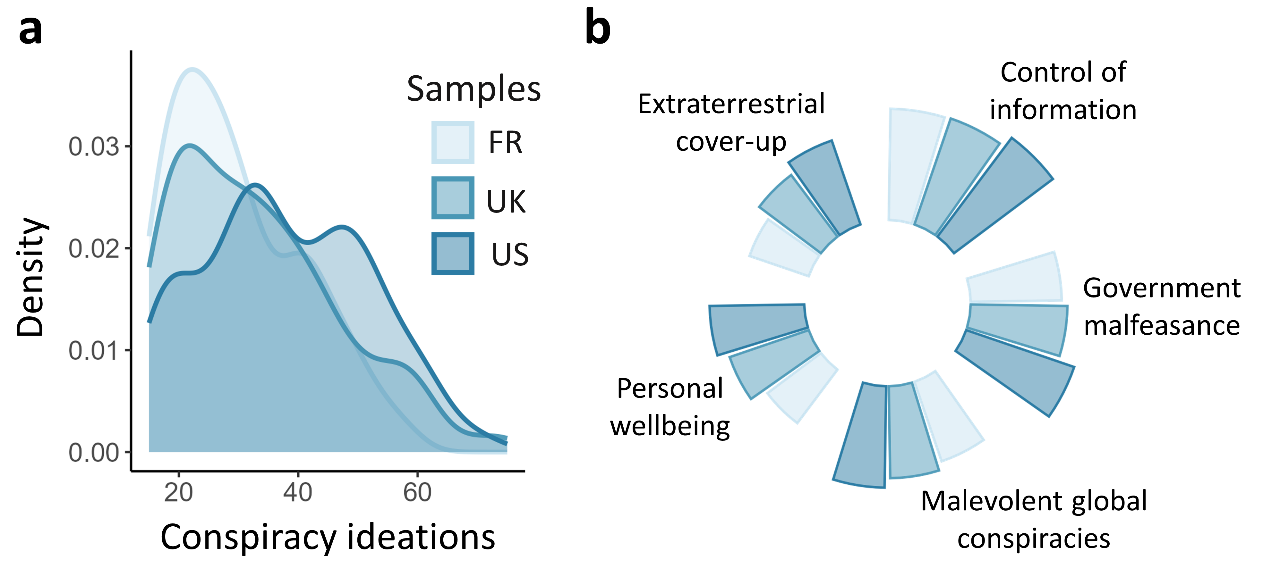


**FIGURE S1. Conspiracy beliefs across tested populations. (a)** Distribution of GCB scores across each national sample i.e., US, *United States of America*; UK, *United Kingdom*; FR, *France*. **(b)** National samples displayed a similar pattern across GCB subscales distribution (control of information, government malfeasance, malevolent global conspiracies, personal well-being and extraterrestrial cover-up).

**TABLE S1. Conspiracy ideations in tested populations at baseline.**

|  | **Control of  information** | **Government malfeasance** | **Malevolent global conspiracies** | **Personal well-being** | **Extraterrestrial cover-up** |
| --- | --- | --- | --- | --- | --- |
| Whole Sample (n = 623) | 8.35 ± 3.10 | 7.28 ± 3.19 | 6.69 ± 3.22 | 5.94 ± 2.85 | 5.53 ± 3.01 |
| US (n = 212) | 8.92 ± 3.12 | 8.23 ± 3.27 | 7.31 ± 3.30 | 6.82 ± 3.21 | 6.40 ± 3.21 |
| UK (n = 225) | 8.08 ± 3.05 | 6.98 ± 3.21 | 6.63 ± 3.40 | 6.02 ± 2.86 | 5.56 ± 3.06 |
| FR (n = 186) | 8.02 ± 3.07 | 6.56 ± 2.82 | 6.06 ± 2.78 | 4.82 ± 2.20 | 4.51 ± 2.33 |

US: United States of America; UK: United Kingdom; FR: France; columns correspond to the 5 subscales of the *Generic Conspiracist Beliefs Scale*.

As mentioned in the *Results* section, we replicated previous findings showing that conspiracy ideations are not normally distributed across nonclinical populations^1^, suggesting that this type of belief is not commonly endorsed by most of the population (**Fig. S1a**). Of note, the scale we used assesses a general degree of adherence to CTs, suggesting that even if a large part of the population could deem certain CTs believable to a certain degree, rigidity of adherence to such beliefs is an isolated phenomenon only represented by an extreme fringe of the tested sample.

Conversely, we did not replicate previous findings suggesting that males were more prone to endorsing CTs^2^. We compared GCB scores between males and females using a Mann-Whitney test and found no significant difference (W = 47832, p = .868, Cohen’s *d* = .0017, **Fig. 3b**). Of note, six participants preferred not to specify their sex and were excluded from this specific analysis (this sample was too small to be considered in itself).

Congruent with the literature, we found that a higher degree of conspiracy endorsement is associated with lower educational attainment. We compared GCB scores according to ISCED levels of education using Welch ANOVA and found a significant difference between the *low*, *medium* and *high* education groups (**F = 13.477, p <.001, η^2^=.395 e^-05^**, **Fig. 3d**). This effect could be partially explained by multiple intertwined factors associated with education, such as analytical thinking and belief in simple solutions for complex problems^3^.

Similarly, we observed an association between a higher degree of conspiracy endorsement and younger age using Welch ANOVA (**F = 3.1015, p = .046, η^2^=.039**, **Fig. 3c**). Initially, we speculated that this effect might be driven by the educational factor mentioned earlier. However, the linear model testing for the age x education interaction only showed a trending variation in GCB scores. Therefore, we argue that education cannot fully account for this relationship, particularly as it does not explain the observed decrease in conspiracy endorsement after 30 years of age, when individuals typically stop pursuing institutional education. This finding contradicts previous results showing a positive relationship between age and belief in conspiracy theories^4^. This discrepancy might be due to a difference in methodology. While the aforementioned authors measured a percentage of believers in specific COVID-19-related CTs in different age groups, we evaluated a general degree of adherence to CTs. Thus, while the tendency to deem health-related CTs believable might increase with age – perhaps due to an increased feeling of health-related threat that have been found associated with conspiracy ideations^5^ – there might be a general tendency for CT adherence to decline over the life course. In particular, this interpretation is in line with existing literature involving a similar approach to measuring conspiracy beliefs^6^.

## Controlling for experimental design biases

### Pilot data

We tested the validity of the online perceptual stability test before the main experiment. An independent sample of 16 participants performed the NC task twice: (i) online and (ii) supervised in a laboratory setting. The order between these two conditions was counterbalanced. Details about the online administration of the task can be found in the *Methods* section. We kept the same design for the in-lab version. We ensured that participants were installed 60 cm from the screen and that their eyes were aligned with the middle of the screen using a chin-strap. They were placed in the dark and received the same instructions as the online sample but were told that they could ask the investigator for further explanations if the instructions were unclear.

We checked for differences in stability scores between the two methods using a Wilcoxon signed-rank test for repeated measures and found no significant difference (W = 92, p = .231, **Fig. 2d**). We then assessed the range of agreement between the two methods using a Bland Altman test and found a nonsignificant mean-difference bias of .0586 (**Fig. 2e**). All individual differences were contained in the *limits of agreement* ([-.300 ; .417]), supporting a good agreement between online and in-lab stability measures in the same participants.

To control for a potential training effect on the perceptual task induced by this pilot repeated-measures design, we checked for changes in stability scores between the first and second assessment in the pilot sample using a Wilcoxon signed rank test for repeated measures. The values at baseline (mean stability score = .474, s.d. = .179) did not differ from those at retest (mean stability score = .467, s.d. = .160 ; W = 67, p = .980) suggesting that any test-retest difference further observed cannot be attributed to training effects.

**FIGURE S2. Perceptual stability in each national sample.**

**
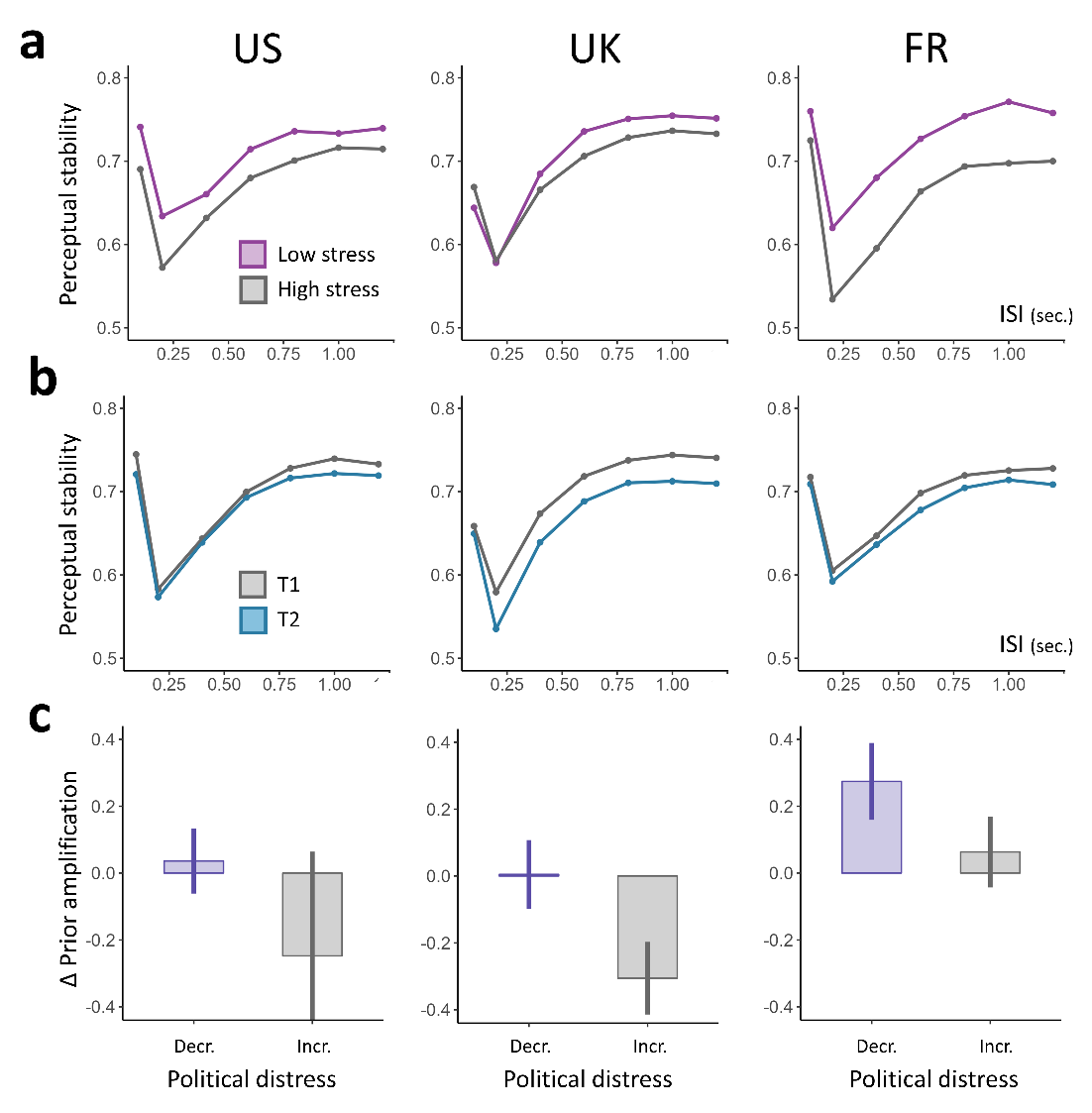
(a)** Perceptual stability as a function of interstimulus interval (ISI) in *the low stress* (LS) and *high stress* (HS) groups across the 3 samples (US: *United States of America*; UK: *United Kingdom* and FR: *France*).

**(b)** Perceptual stability as a function of interstimulus interval length over time across the 3 samples (T1: baseline; T2: retest).

**(c)** evolution of prior amplification (ΔPrior) over time for the ‘Dec’ and ‘Inc’ groups across the 3 samples (Dec: decreased stress; Inc: increased stress).

We added attentional checks during the psychometric assessment to ensure participants did not provide random answers. Among the scales used, five items were randomly added, regularly asking participants for a specific answer (example: “*This is an attentional check, please answer ‘Not sure/cannot decide’ to that question.*”).

### Experimental samples

To further strengthen the validity of our method, we compared stability scores at baseline between the three national samples (US, UK and FR) using Welch's ANOVA and found no significant difference (F(2,620) = .81828, p = .4419). While CTs were significantly greater in the US sample (**F(2,412) = 19.038, p < .001, η² = 3.477967e^-8^**), we observed a similar pattern across the GCB subscale distribution of the three tested samples (**Fig. S1b**). Furthermore, we observed the same patterns of association between (i) political distress at baseline and perceptual rigidity (**Fig. S2a**) and (ii) a decrease in perceptual stability between the first and second measurements (**Fig. 2c, Fig. S2b**). Finally, we investigated whether the uncertainty induced by the bistable task could influence adherence to conspiracy beliefs or whether the activation of conspiracy ideations prior to the behavioral task could affect perceptual stability. We administered the NC task and the self-reported assessment of beliefs in a randomized order to control for those biases. Moreover, we compared both groups of participants randomly assigned to an order using Mann‒Whitney tests and found no difference in perceptual stability (U = 47550, p = .767) or GCB scores (U = 45526, p = .230).

To ensure that this phenomenon of global destabilization was specifically associated with the dynamics of sociopolitical uncertainty, we tested stability a third time on the UK and FR samples one month after the second acquisition (**Fig. 2c**). We compared stability at baseline and during the third test using Wilcoxon signed-rank tests for repeated measures. We found no significant difference whether we tested the whole sample (W = 30618, p = .141) or the 3 national subsamples independently (UK: W = 9952, p = .247 ; FR: W = 5729, p = .137), excluding a possible learning effect across the sessions.

## Self-reported measures

At each time step, participants were instructed to rate their level of distress related to the ongoing event in their country using 10-point visual analogue scales. Political distress scores were obtained by computing the mean of these ratings. We asked the following questions:

*Political distress assessment at baseline:*

- US : How distressed were you during the week preceding the 2020 presidential elections?
- UK : How distressed are you since the announcement of Brexit?
- UK : How distressed are you regarding the establishment of BREXIT that will come to pass?
- UK : How distressed are you regarding the consequences of Brexit?
- UK : How distressed are you regarding the possibility of a no-deal?
- FR : À quel point êtes vous stressé(e) par l’approche des élections présidentielles ?
- FR : À quel point êtes vous stressé(e) par les conséquences de l’élection présidentielle à venir ?

*Political distress assessment at retest:*

- US : How distressed are you since the announcement of the 2020 presidential election outcome in the media?
- UK : How distressed are you since the UK left the EU?
- UK : How distressed are you regarding the consequences of Brexit?
- FR : À quel point êtes vous stressé(e) par le résultat des élections présidentielles ?
- FR : À quel point êtes vous stressé(e) par les conséquences de l’élection présidentielle ?

## The Circular Inference parameters

The Circular Inference model (CI) facilitates the extraction of four parameters contributing to the perceptual decision of each individual subject: the weight of the sensory gain (**sensory**), the amplification or prior beliefs (**prior**), the strength of the bias (**bias**), and a fourth parameter **(penalty**; see also the ***Methods*** section of the main manuscript). These four parameters are computed based on the perceptual behavior of participants (**Fig. S3e**), which is experimentally measured through perceptual stability scores and follows a reversed U-shaped distribution (**Fig. S3a, b, c, d**).

**
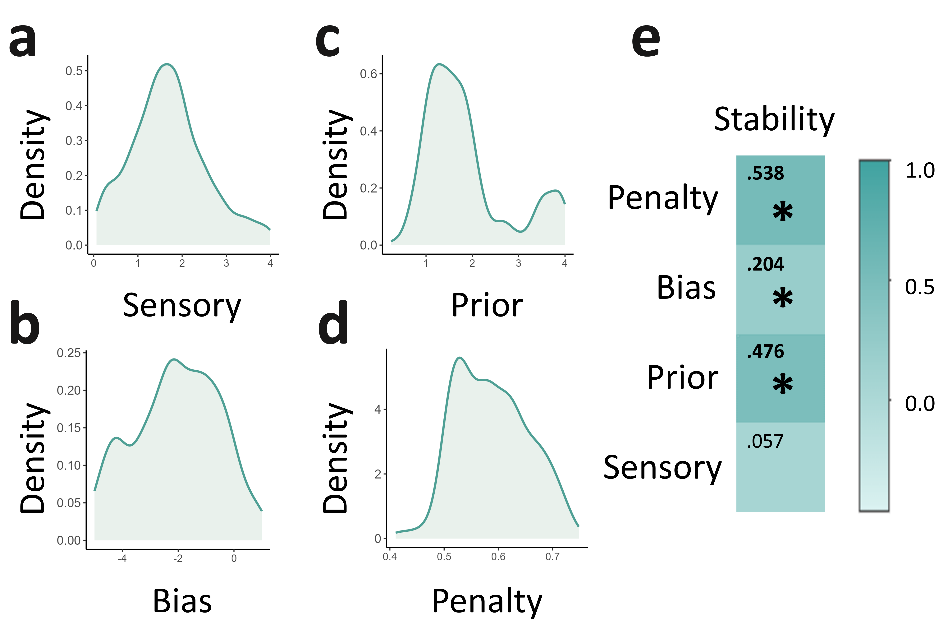
Figure S3. The Circular Inference (CI) model parameters and their relation to the perceptual stability**.

Distribution of CI parameters in the entire sample, that respectively corresponds to **(a)** the sensory ; **(b)** the bias ; **(c)** the prior ; **(d)** the penalty. **(e)** Heatmap depicting the strength of associations at baseline between the CI parameters and perceptual stability (Spearman’s correlations, corrected for multiple comparisons using the *false discovery rate* method, FDR).

Parameter recovery was performed by randomly selecting 200 subject/session combinations. Using the CI parameter fits, we generated new responses for 200 simulated subjects by running the model a single time on a random sequence of OFF durations (generated the same way we did for the actual experiment) with the same number of trials. We then reran the optimization procedure on the simulated data to ensure that the fitted parameters were close to the parameters used to generate the data (**Fig. S4**). Additionally, a one-way intraclass correlation test (ICC) was performed between the second and third time points (T2 and T3, respectively) to check for acceptable test-retest reliability of the CI parameters. Despite a limited correlation strength, we demonstrated significance for each parameter (penalty: **ICC = .471, p < .001**; bias: **ICC = .271, p < .001**; prior: **ICC = .408, p < .001**; sensory: **ICC = .169, p < .001**).

**Figure S4. Parameter Recovery of the Circular Inference (CI) model**.


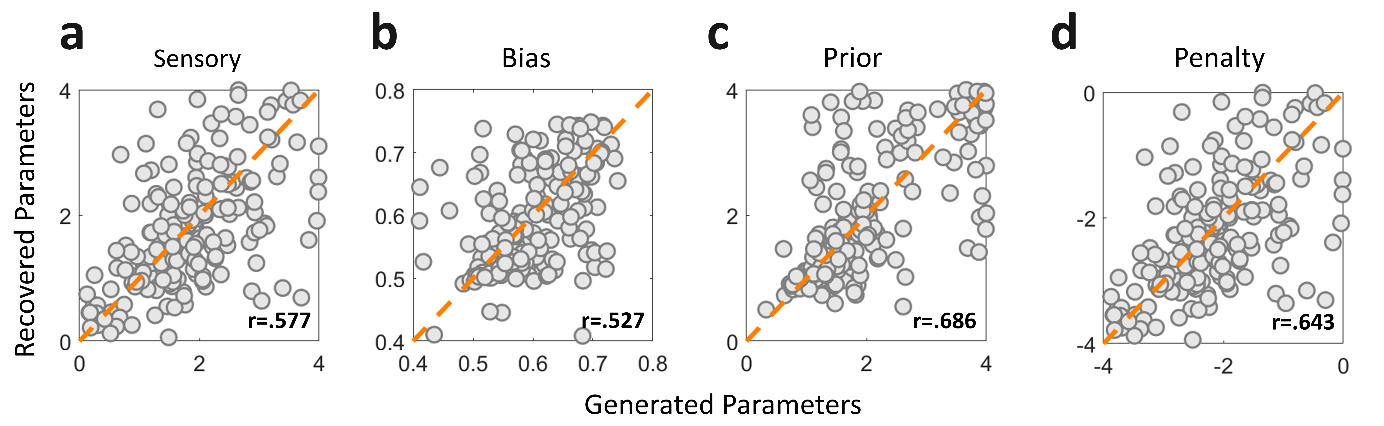


Scatter plots showing the good concordance (above 0.5) between generated and recovered parameters with CI using 200 random participants among the 649 enrolled. Spearman’s rho coefficients are provided at the bottom of the plots. Significance for: **(a)** sensory gain : p < .001 ; **(b)** Bias for the dominant percept : p < .001 ; **(c)** Prior amplification : p < .001 ; **(d)** Penalty (ON/OFF volatility): p < .001.

## Supplementary figures

**Figure S5. Evolution of the Circular Inference parameters over time according to stress dynamics.**
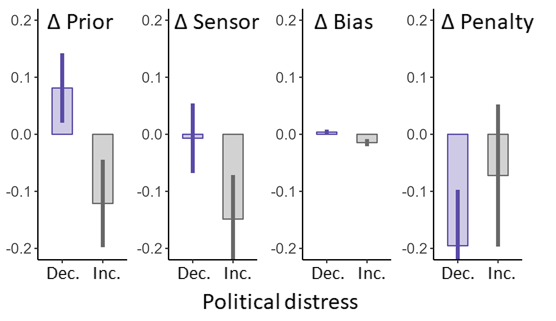


Some participants demonstrated decreased political distress after uncertainty resolution (Dec, n = 330) while others showed increased stress in the same period (Inc, n = 330). From left to right: evolution of prior amplification (ΔPrior) over time for ‘Dec’ group (mean = .081, s.d. = 1.11) and ‘Inc’ group (mean = -.121, s.d. = 1.14); evolution of sensory overweighting (ΔSensor) over time for the ‘Dec’ group (mean = -.007, s.d. = 1.11) and ‘Inc’ group (mean = -.149, s.d. = 1.15); evolution of bias (ΔBias) over time for the ‘Dec’ group (mean = .004, s.d. = .079) and ‘Inc’ group (mean = -.015, s.d. = .093); evolution of volatility (ΔPenalty) over time for the ‘Dec’ group (mean = .195, s.d. = 1.78) and ‘Inc’ group (mean = -.072, s.d. = 1.85).

**FIGURE S6. The shift in the balance of Circular Inference parameters is associated with the evolution of conspiracy ideations after the resolution of uncertainty.**


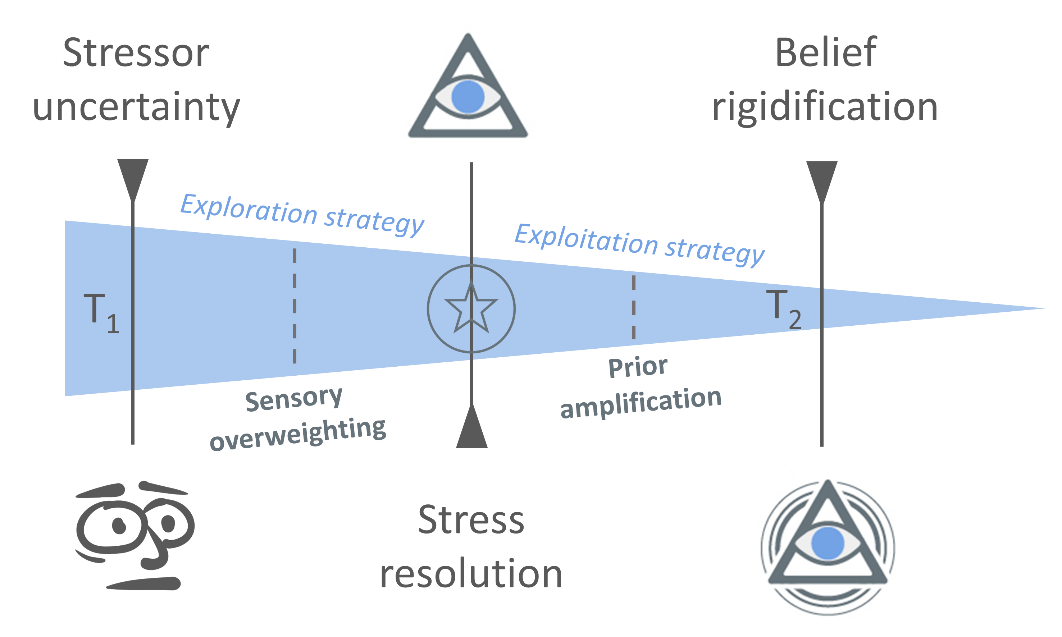


A possible hypothesis is that, motivated by the need to cope with uncertainty, distressed participants first adopt an “exploration” strategy, seeking simple and intuitive explanations in their environment to make their perceptual decisions; they secondarily shift to an “exploitation” strategy, validating their newly established view and reinforcing their own beliefs.

## Supplementary tables

**TABLE S3. Conspiracy and Circular Inference parameters over time according to stress dynamics.**

|  | **ΔGCB** | **Δsensory** | **Δprior** | **Δbias** | **Δpenalty** |
| --- | --- | --- | --- | --- | --- |
| Dec (n = 330) | 1.29 ± 10.28 | -.01 ± 1.11 | .08 ± 1.11 | .004 ± .08 | .19 ± 1.78 |
| Inc (n = 220) | -.31 ± 11.61 | -.15 ± 1.15 | -.12 ± 1.14 | -.02 ± .09 | -.07 ± 1.85 |

Dec, ‘Dec’ group corresponding to participants who decreased their stress over time; Inc: ‘Inc’ group corresponding to participants who increased their stress over time; ΔGCB: evolution in *Generic Conspiracist Beliefs Scale* over time; Δsensory: evolution of sensory overweighting over time; Δprior: evolution of prior amplification over time; Δpenalty: evolution of volatility over time; Δbias: evolution of bias over time. (see also ***Methods***).

# **REFERENCES**

1. Bronstein, M. V., Kummerfeld, E., MacDonald, A. & Vinogradov, S. Willingness to vaccinate against SARS-CoV-2: The role of reasoning biases and conspiracist ideation. *Vaccine* **40**, 213–222 (2022).

2. Freeman, D. & Bentall, R. P. The concomitants of conspiracy concerns. *Soc. Psychiatry Psychiatr. Epidemiol.* **52**, 595–604 (2017).

3. van Prooijen, J.-W. Why Education Predicts Decreased Belief in Conspiracy Theories. *Appl. Cogn. Psychol.* **31**, 50–58 (2017).

4. Romer, D. & Jamieson, K. H. Conspiracy theories as barriers to controlling the spread of COVID-19 in the U.S. *Soc. Sci. Med. 1982* **263**, 113356 (2020).

5. Federico, C. M. & Malka, A. The contingent, contextual nature of the relationship between needs for security and certainty and political preferences: Evidence and implications. *Polit. Psychol.* **39**, 3–48 (2018).

6. Wagner-Egger, P., Bangerter, A., Delouvée, S. & Dieguez, S. Awake together: Sociopsychological processes of engagement in conspiracist communities. *Curr. Opin. Psychol.* **47**, 101417 (2022).
